# miRNAs Are Involved in Determining the Improved Vigor of Autotetrapoid *Chrysanthemum nankingense*

**DOI:** 10.3389/fpls.2016.01412

**Published:** 2016-09-28

**Authors:** Bin Dong, Haibin Wang, Aiping Song, Tao Liu, Yun Chen, Weimin Fang, Sumei Chen, Fadi Chen, Zhiyong Guan, Jiafu Jiang

**Affiliations:** ^1^Lab of Chrysanthemum Genetics, Breeding and Molecular Biology, College of Horticulture, Nanjing Agricultural UniversityNanjing, China; ^2^Jiangsu Province Engineering Lab for Modern Facility Agriculture Technology and EquipmentNanjing, China

**Keywords:** microRNA, autopolyploidization, vigor, growth and development, adaptation

## Abstract

Many plant species are autopolyploid, a condition frequently associated with improvements in both vegetative and reproductive vigor. The possible contribution of miRNAs to this improvement was investigated by characterizing the miRNA content of a diploid and an autotetraploid form of *Chrysanthemum nankingense*. 162 and 161 known miRNA sequences were identified in 2x and 4x library. The length of 22 and 25 nt was predominant in diploid. However, 21 and 24 nt showed dominance in autotetraploid. It seems likely that autopolyploidization have had an immediate effect the distribution of miRNAs. In addition, the abundance of the miRNAs differed markedly between the two ploidy levels and contributed to their targets diversity. A number of target genes associated with miRNAs play important roles in growth and development. The conclusion was that some miRNAs likely make a contribution to the vigor displayed by autotetraploid *C. nankingense*.

## Introduction

Polyploidization is an important evolutionary driver in the plant kingdom, and provides plants with a means to adapt rapidly to a new environmental niche and to speciate (Otto, 2007; Ainouche and Jenczewski, 2010; Wang et al., 2013a). It has been estimated that at least 70% of angiosperm species have undergone at least one polyploidization event during their evolution (Masterson, 1994; Soltis and Soltis, 1999). While autopolyploids represent the doubling of a given species' chromosome complement, allopolyploids derive via the chromosome doubling of a hybrid between sexually compatible, but non-identical species. Once such an event has occurred, a number of both genetic and epigenetic modifications are typically induced (Comai, 2000; Osborn et al., 2003; Chen, 2007; Wang et al., 2014). These occur rather frequently in *de novo* allopolyploids, while in autopolyploids, only a limited amount of genomic reorganization appears to be induced (Rieseberg and Willis, 2007).

The class of small (20–24 nt) non-coding RNA molecules referred to as microRNAs (miRNAs) are generated from a stem-loop structure precursor. Their biological significance lies in their ability to regulate gene expression by targeting a specific transcript for degradation (Bartel, 2004; He and Hannon, 2004). A number of miRNAs are known to play an important role in plant growth and development (Rubio-Somoza et al., 2009; Cao et al., 2016) and in the response to biotic and abiotic stress (Ruiz-Ferrer and Voinnet, 2009; Gao et al., 2016). Furthermore, miRNAs can affect gene expression by reducing stochastic noise, buffer cross-species variation and constrain evolutionary gene expression variation (Cui et al., 2007). In allopolyploids involving *Arabidopsis thaliana*, certain miRNAs have been shown to act as a buffer against the genomic shock experienced by *de novo* interspecific allopolyploids (Ha et al., 2009). So far, their effect in autopolyploids has received little attention.

The diploid species *Chrysanthemum nankingense* is a native of China (Wang et al., 2013b; Sun et al., 2014). Just as polyploids are frequently phenotypically superior to their lower ploidy progenitor (Otto, 2007), a number of the organs formed by autotetraploid *C. nankingense* are significantly larger than those of the diploid form (Liu et al., 2011). Given the important roles played by miRNAs in regulating plant growth and development in polyploid plants, a program was initiated to contrasting the miRNA content of the diploid and the autotetraploid, with a view both to revealing how polyploidization influenced the content and distribution of miRNAs, and to exploring whether miRNA activity could be responsible for the improved vigor of the autotetraploid.

## Materials and methods

### Plant materials and growing conditions

Diploid (2*n* = 2*x* = 18) and autotetraploid *C. nankingense* (2*n* = 4*x* = 36) plants were raised in a greenhouse under a ~16 h photoperiod, a constant temperature of ~25°C and a relative humidity of 70–75%. The materials were sourced from the Nanjing Agricultural University Chrysanthemum Germplasm Resource Preserving Centre. At the vegetative state (10–15 leaf old), measurements were made of plant height, leaf length, leaf width, leaf area, and stem length using caliper (Mitutoyo, Japan). Leaf area was estimated from digital images using ImageJ software (Schneider et al., 2012).

### RNA extraction and small RNA (sRNA) sequencing

Plants were grown up to the 10–15 leaf stage, at which point leaf and stem was harvested from 3–5 plants. Total RNA was isolated from 0.5 g plant tissue using the RNAiso Plus reagent (TaKaRa, Dalian, China) then treated with RNase-free DNase I (TaKaRa) to remove contaminating DNA. A 5 μg aliquot of pooled RNA was obtained by combining, on an equimolar basis, the RNA extracted from three biological replicates. The quality and production of the RNA were detected using, respectively, an Aglilent 2100 Bioanalyzer (Agilent Technologies, Palo Alto, CA, USA) and the ABI Step One Plus-Time PCR System (Applied Biosystems, Foster City, CA, USA). The sequencing of the sRNA fraction was carried out on an Illumina 2000 device housed at the Beijing Genomics Institute (http://www.genomics.cn). The datasets are available in the NCBI repository, (http://trace.ncbi.nlm.nih.gov/Traces/sra_sub/sub.cgi?) under accession number SRP080104. Before analysis, adapter sequences, 10% of the nitrogen sequences and low quality (*Q* < 5) reads were removed, leaving two separate sRNA libraries of “clean” reads (one from diploid *C. nankingense* and the other from the autotetraploid form). Then the length distribution of clean reads, total, and unique sequences was summarized.

### miRNA annotation and target gene prediction

The set of sRNAs (17–30 nt) represented in the two libraries were filtered to remove ribosomal RNAs, transfer RNAs, small nucleolar RNAs and small nuclear RNAs, and the remaining sequences were then compared with equivalent plant non-coding sRNA sequences deposited in either the GenBank (http://www.ncbi.nlm.nih.gov/genbank/) or the Rfam (http://www.sanger.ac.uk/science/tools/rfam) database. The set of filtered sRNA sequences were aligned via BlastN with those represented in the miRNA database (http://www.mirbase.org). Because the miRNA data of chrysanthemum is absent in miRbase, the general criteria is that small RNAs align to the precursor or mature sequences of corresponding species and obtain miRNA count in miRbase. The novel miRNAs were predicted using software Mireap (http://sourceforge.net/projects/mireap/). The copy number of individual miRNAs was expressed in the form of reads per million reads (RPM), and the criterion set for differential abundance between the two libraries was that the log_2_ of the ratio between the two RPMs was either >+1 or < −1, along with *P*-value of < 0.001. To enable this, miRNAs with an RPM of zero were assigned a nominal RPM of 0.01. Targets for each selected *C. nankingense* miRNA were defined by using its sequence as a query term against the *C. indicum* transcriptome described by Wang et al. (2013b) and lodged in GenBank as accession number PRJNA245057. Target prediction was based on the Allen et al. (2005) criteria using psRNATarget server (Dai and Zhao, 2011). The predicted targets were functionally classified following the Gene Ontology (http://www.geneontology.org) method, and assigned a pathway using the KEGG database (Kanehisa et al., 2016; http://www.genome.jp/kegg/kegg1.html).

### Poly (A) tailing and reverse transcription

Poly (A) tailing and reverse transcription were performed using a PrimeScript® miRNA qPCR Starter Kit v2.0 (TaKaRa), following the manufacturer's protocol. Briefly, each 20 μL reaction contained 10 μL 2 × miRNA Reaction Buffer Mix, 2 μL 0.1% w/v BSA, 2 μL miRNA PrimeScript® RT Enzyme Mix, 1 μL total RNA, and 5 μL RNase-free distilled water. Total RNA were isolated from plant tissue of independent plant materials at the 10–15 leaf stage. The Poly (A) tailing reactions and reverse transcription were incubated at 37°C for 2 h, then inactivated by holding at 85°C for 5 s.

### Real time quantitative-PCR (qRT-PCR)

Each 20 μL qRT-PCR contained 10 μL SYBR® *Premix Ex Taq*™ II (Takara), 5 μL cDNA (80 ng/μL), 1.0 μL 10 μM forward primer (designed using primer 5.0 software), and 1.0 μL Uni-miR qPCR Primer. The reactions were held at 95°C for 2 min, then subjected to 40 cycles of 95°C/15 s, 55°C/15 s, and 72°C/20 s. The chrysanthemum gene *EF1*α (Genbank accession number KF305681) was used as the reference sequence, following the suggestion of Wang et al. (2015). In addition, three biological replicates and three technical replicates were performed for each tissue sample. Amplicon abundances were calculated using the 2^−ΔΔCT^ method (Livak and Schmittgen, 2001).

## Results

### Phenotypic effect of autopolyploidization

**Figure 1:**
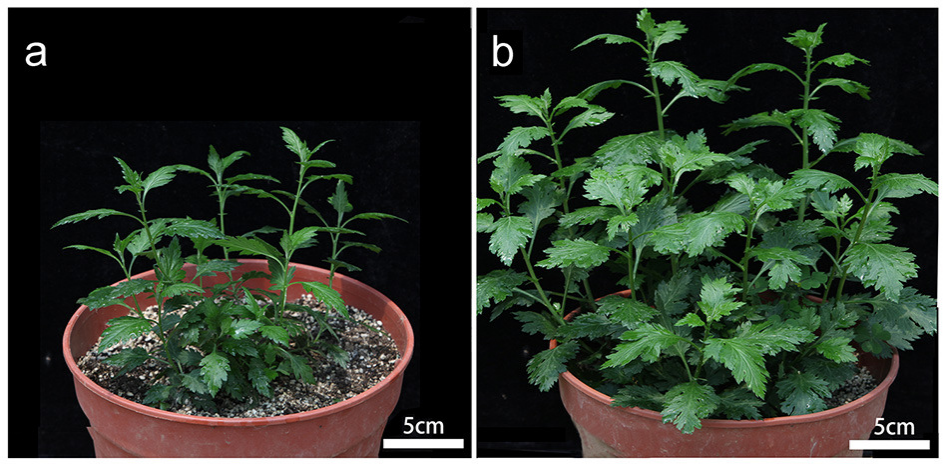
**The improved vigor displayed by the autotetraploid form of *C. nankingense*. (A)** Diploid plant, **(B)** autotetraploid plant. In both cases the plants had developed 10–15 leaves.

As also noted by Liu et al. (2011), the tetraploid form of *C. nankingense* was phenotypically distinct from the diploid form (Figure 1). In particular, plant height, leaf length, and leaf area were all greater in the autotetraploid than in the diploid at vegetative stage (Table 1). These results imply that autotetraploid plants were more vigorous than the diploid, and higher overall biomass was generated during the growth of autotetraploid.

**Table 1:** **Differences in the morphology of the diploid and autotetraploid forms of *C. nankingense***.

	**Plant height (cm)**	**Leaf length (cm)**	**Leaf width (cm)**	**Leaf area (cm^2^)**	**Stem length (cm)**
Diploid	7.98 ± 0.36	4.87 ± 0.16	2.63 ± 0.08	4.12 ± 0.45	1.08 ± 0.054
Autotetraploid	13.9 ± 0.53^*^	6.50 ± 0.17^*^	3.54 ± 0.13	9.61 ± 0.54^*^	1.17 ± 0.044

*Asterisks represent significant difference between the diploid and autotetraploid according to Student's test (p < 0.05)*.

### The miRNA content of diploid and autotetraploid *C. nankingense*

**Figure 2:**
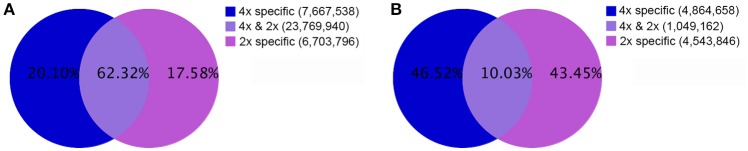
**The population of sRNAs in diploid and autotetraploid *C. nankingense*. (A)** The set of total sequences, and **(B)** the set of unique sequences in diploid and autotetraploid library.

**Figure 3:**
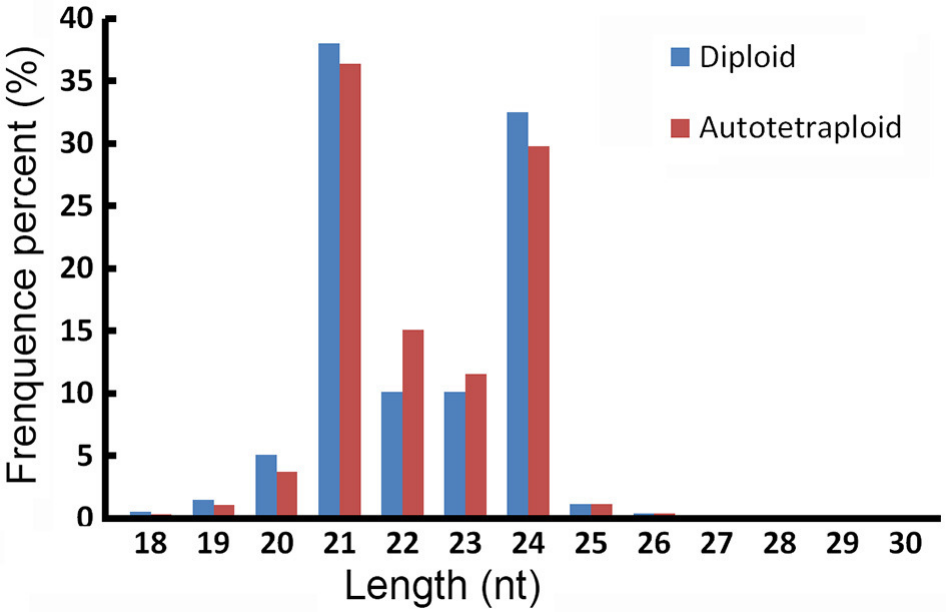
**The length distribution of sRNAs in 2x and 4x *C. nankingense***.

In all 18,217,460 raw reads were acquired from 2x *C. nankingense* and 20,864,890 from the 4x form (Table 2). The filtering step reduced these numbers to, respectively, 17,984,240 and 20,561,513; of these, 98.77% were classified as clean reads in the 2x library and 99.11% in the 4x one. The combined set of 38,141,274 clean reads included 10,457,666 unique sequences (Figure 2, Table S1). Then total sRNAs and unique sRNAs aligned the NCBI GenBank (Table 3). Except miRNAs, many types of RNA sequences including other non-coding RNAs (tRNAs, rRNAs, snoRNAs, and snRNAs etc.), sRNAs of unknown origin and degraded fragments were removed (Table S2). In addition, the length range of the small RNAs were summarized in Figure 3, the reads of 21 and 24 nt long was abundant in the 2x and 4x library.

**Table 2:** **The number of sequencing reads obtained from the diploid and autotetraploid forms of *C. nankingense***.

**Category**	**Diploid**		**Autotetraploid**	
	**Count**	**Percentage (%)**	**Count**	**Percentage (%)**
Raw reads	18,217,460		20,864,890	
High quality	17,984,240	100	20,561,513	100
Clean reads	1,7762,959	98.77	20,378,315	99.11
3′adapter null	82,633	0.46	102,925	0.50
Insert null	5453	0.03	1928	0.01
5′adapter contaminants	31,204	0.17	29,241	0.14
Smaller than 18nt	100,429	0.56	47,865	0.23
Poly A	1562	0.01	1239	0.01

**Table 3:** **Analysis of sequencing reads mapping of sRNA in diploid and autopolyploid library of *C. nankingense***.

**Library**	**Total reads**	**Unique reads**	**Total RNAs mapped to genome**	**Unique RNAs mapped to genome**
Diploid	17,762,959	5,593,008	7,264,633 (40.9%)	605,570 (10.83%)
Autotetraploid	20,378,315	5,913,820	8,797,776 (43.17%)	690,383 (11.67%)

**Figure 4:**
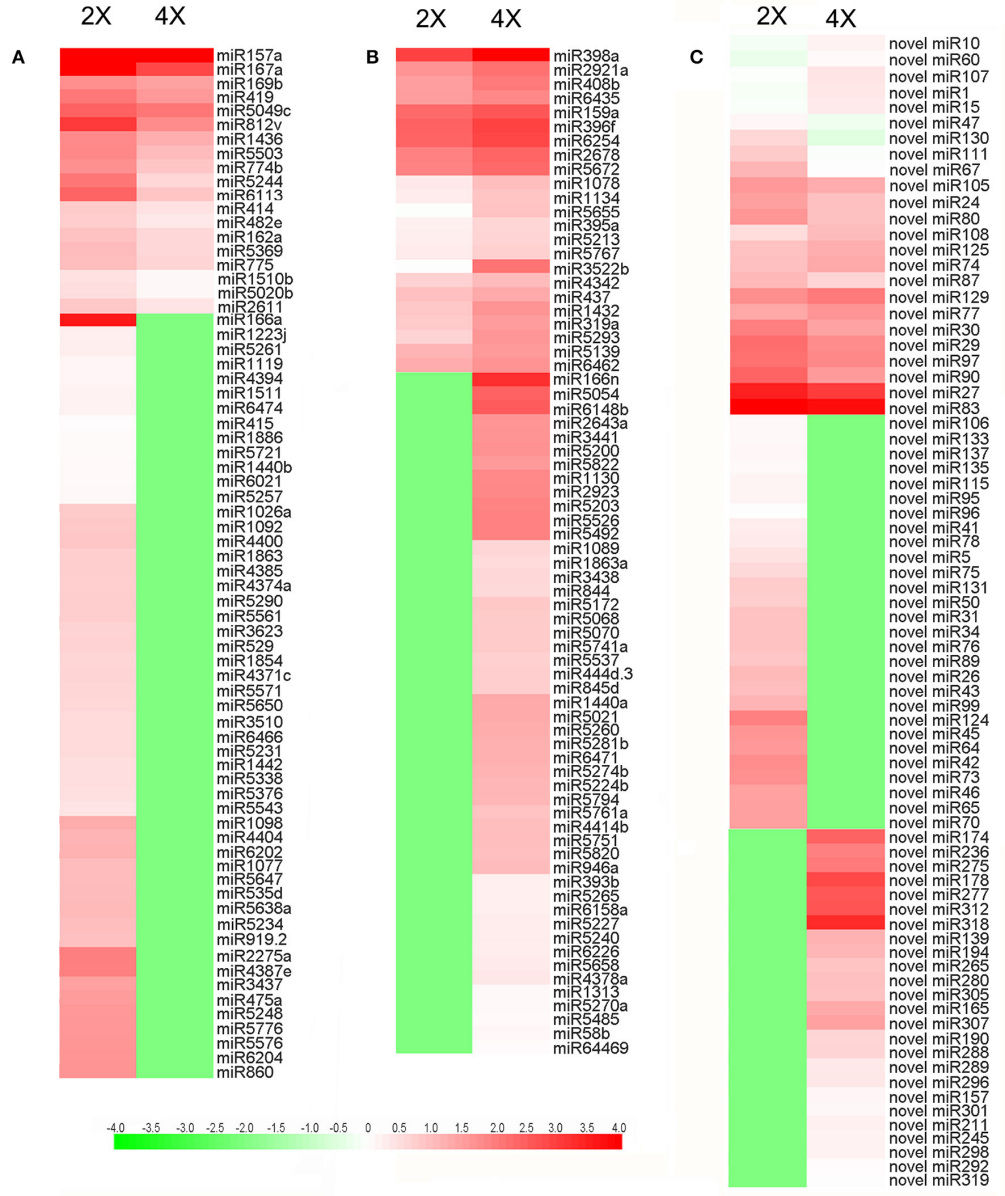
**Differential abundance of miRNAs in 2x and 4x *C. nankingense*. (A,B)** Known miRNAs, **(C)** novel miRNAs. The color intensity reflects the magnitude of the logarithm of the abundance.

A comparison with the sequences represented in miRBase revealed that the 2x and 4x libraries included, respectively, 162 and 161 known miRNA sequences (Table S3). Based on the criteria applied for differential abundance, 72 miRNAs were classed as significantly more abundant in the 4x form and 71 significantly less abundant, their relative abundances are illustrated in Figures 4A,B. Meanwhile 49 miRNAs were present in the 4x form but were down-regulated in the 2x form, and 52 *vice versa* (Table S4). 61 and 65 novel miRNAs was identified in 2x and 4x library (Table S5). Of these, 28 were down-regulated in the 4x form but were present in the 2x form and 25 *vice versa* (Table S6). The differentially abundant showed in Figure 4C, with 14 miRNAs suppressed and 10 miRNAs enhanced in the 4x form.

### Predicted miRNA targets

**Figure 5:**
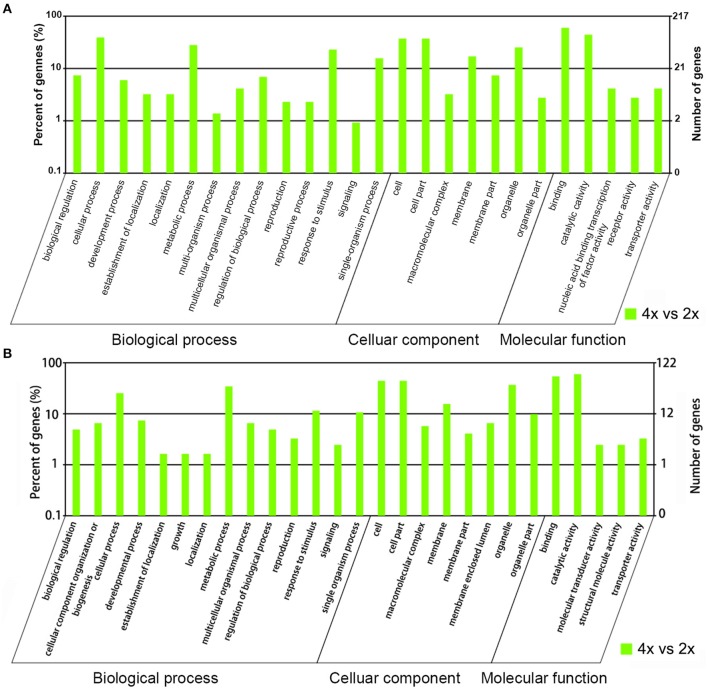
**Gene ontology of differentially abundant miRNAs**. The targets of **(A)** known miRNAs, and **(B)** novel miRNAs which were more abundant in the 4x than the 2x form of *C. nankingense*.

The potential targets of the miRNAs were predicted from the *C. indicum* transcriptome, along with the potential cleavage sites (Sheet 1). For the known miRNAs scored as differentially abundant in the 4x form, 531 potential targets were associated with 78 miRNAs, and were classified into 26 functional Gene Ontology groups (Sheet 2). The predominant biological process was “cellular process,” that of the cellular component was “cell and cell part” and that of function was “binding and catalytic activity” (Figure 5A, Sheet 2). Reference to the KEGG database allowed 302 of the targets to be assigned to 115 pathways (Sheet 3). With respect to 70 novel miRNAs which showed different abundance in the 4x form, 622 potential targets were identified and classified into 27 functional groups (Sheet 2). The predominant biological process was “metabolic process,” that of the cellular component was “cell, cell part, and organelle” and that of function was “binding and catalytic activity” (Figure 5B, Sheet 2). Reference to the KEGG database allowed 187 of the targets to be assigned to 93 pathways (Sheet 3).

### Targets involved in growth- and development-related miRNAs

With respect to genes involved in plant growth and development, there were 45 targets among the ones associated with the set of miRNAs scored as more abundant in the 4x than in the 2x form of *C. nankingense* (Table 4). Three of the novel miRNAs targeted genes of this type: One was a homolog of a gene encoding FLO/LFY-like protein targeted by novel-miR97, the second encoded an EIN4 homolog (ethylene receptor) targeted by novel-miR130, and the third was a MYB transcription factor (MYB46) targeted by novel-miR211 (Table 4).

**Table 4:** **Potential target genes involved in growth and development of miRNAs in the 2x and 4x form of *C. nankingense***.

**miRNA**	**Target gene ID**	**Target gene**	**Annotation**
**KNOWN MIRNA**			
miR156/157a	CL5834.Contig2_All	*SPL13*	Squamosa promoter-binding-like 13
	Unigene12160_All	*SPL3*	Squamosa promoter-binding-like 3
	Unigene1274_All	*SPL18*	Squamosa promoter-binding-like 18
	Unigene3384_All	*SPL16*	Squamosa promoter-binding-like 16
	Unigene5503_All	*SPL10*	Squamosa promoter-binding-like 10
miR160a	CL6917.Contig2_All	*ARF10*	Auxin response factor 10
	CL6948.Contig1_All	*ARF16*	Auxin response factor 16
	Unigene13960_All	*ARF18*	Auxin response factor 18
	Unigene29184_All	*ARF18*	Auxin response factor 18
miR164c	CL12385.Contig1_All	*NAC100*	NAC domain-containing protein 100
	CL17795.Contig2_All	*NAC021*	NAC domain-containing protein 21/22
	Unigene3214_All	*NAC100*	NAC domain-containing protein 100
	Unigene17027_All	*NAC100*	NAC domain-containing protein 100
	Unigene3134_All	*NAC100*	NAC domain-containing protein 100
miR166a	CL2716.Contig5_All	*REV*	Homeobox-leucine zipper protein REVOLUTA
	CL34.Contig3_All	*REV*	Homeobox-leucine zipper protein REVOLUTA
	CL34.Contig4_All	*REV*	Homeobox-leucine zipper protein REVOLUTA
	CL34.Contig5_All	*REV*	Homeobox-leucine zipper protein REVOLUTA
	CL34.Contig6_All	*REV*	Homeobox-leucine zipper protein REVOLUTA
	CL34.Contig7_All	*REV*	Homeobox-leucine zipper protein REVOLUTA
	CL34.Contig8_All	*REV*	Homeobox-leucine zipper protein REVOLUTA
	CL34.Contig9_All	*REV*	Homeobox-leucine zipper protein REVOLUTA
	CL872.Contig8_All	*REV*	Homeobox-leucine zipper protein ATHB-15
miR167a	CL6255.Contig11_All	*ARF8*	Auxin response factor 8
	CL6255.Contig2_All	*ARF8*	Auxin response factor 8
	CL6255.Contig3_All	*ARF8*	Auxin response factor 8
	CL6255.Contig7_All	*ARF8*	Auxin response factor 8
miR168a	Unigene2364_All	*AGO1*	Protein argonaute 1
	CL2282.Contig2_All	*AGO1*	Protein argonaute 1
miR171b	CL239.Contig10_All	*SCL6*	Scarecrow-like protein 6
	Unigene23736_All	*SCL6*	Scarecrow-like protein 6
miR172a	CL11207.Contig2_All	*AP 2*	Transcription factor APETALA 2
	CL17146.Contig2_All	*RAP2–7*	Ethylene-responsive transcription factor RAP2-7
	CL2267.Contig5_All	*AP 2*	Transcription factor APETALA 2
	CL540.Contig3_All	*AP 2*	Transcription factor APETALA 22
miR393b	CL264.Contig2_All	*TIR1*	TRANSPORT INHIBITOR RESPONSE 1
	CL6931.Contig2_All	*TIR1*	TRANSPORT INHIBITOR RESPONSE 1
	Unigene25326_All	*TIR1*	TRANSPORT INHIBITOR RESPONSE 1
miR419	Unigene3306_All	*UPL6*	E3 ubiquitin-protein ligase UPL6
	CL8729.Contig2_All	*UPL6*	E3 ubiquitin-protein ligase UPL6
	CL8218.Contig2_All	*UPL6*	E3 ubiquitin-protein ligase UPL6
miR482e	CL2783.Contig2_All	*bHLH66*	Transcription factor bHLH66
	CL73.Contig2_All	*bHLH66*	Transcription factor bHLH66
miR1098	CL1733.Contig16_All	*UPL1*	E3 ubiquitin-protein ligase UPL1
**NOVEL MIRNA**			
Novel-miR97	Unigene50591_All	*FLO/LFY-like*	FLO/LFY-like protein gene
novel-miR130	CL12185.Contig5_All	*EIN4*	Ethylene receptor EIN4
novel-miR211	CL65.Contig83_All	*MYB46*	Transcription factor MYB46

### qRT-PCR validation of the differential abundance of selected miRNAs and their targets

**Figure 6:**
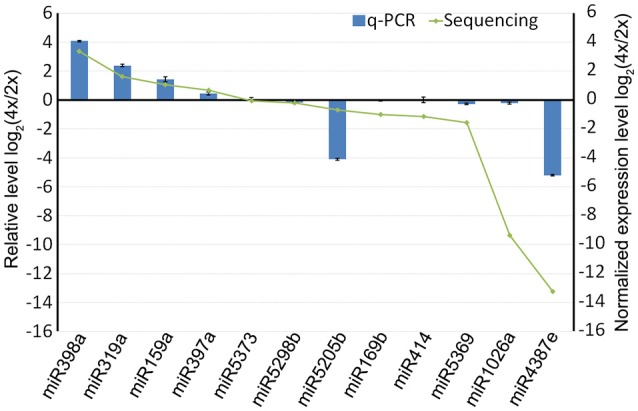
**The abundance of selected miRNAs derived from a qRT-PCR experiment compared to the predicted outcome from the sequencing data**. Each bar shows the mean ± SE of a triplicated assay.

**Figure 7:**
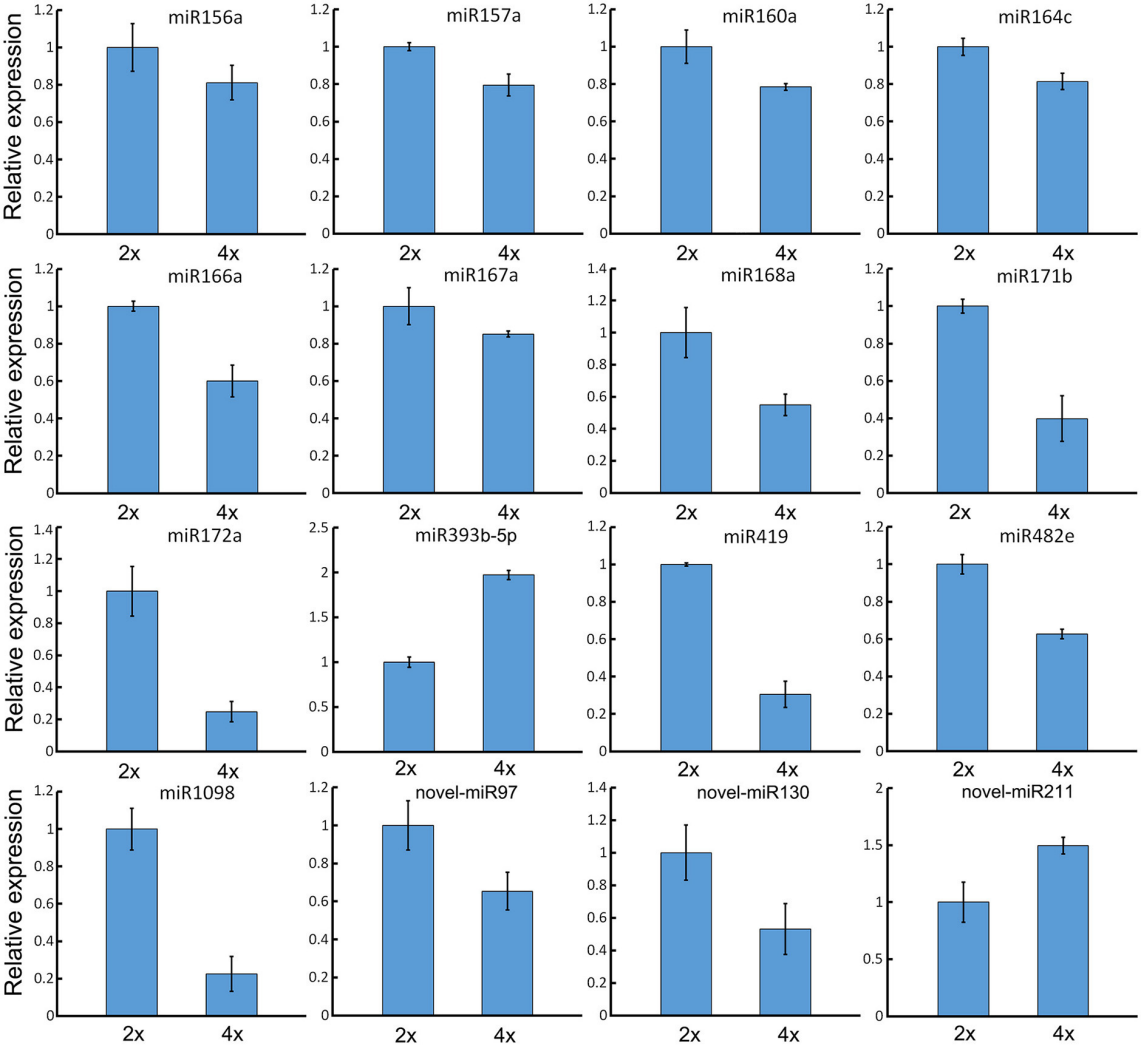
**Validation of expression patterns of miRNAs involved in growth and development by qRT-PCR**. Each bar shows the mean ± SE of a triplicated assay.

**Figure 8:**
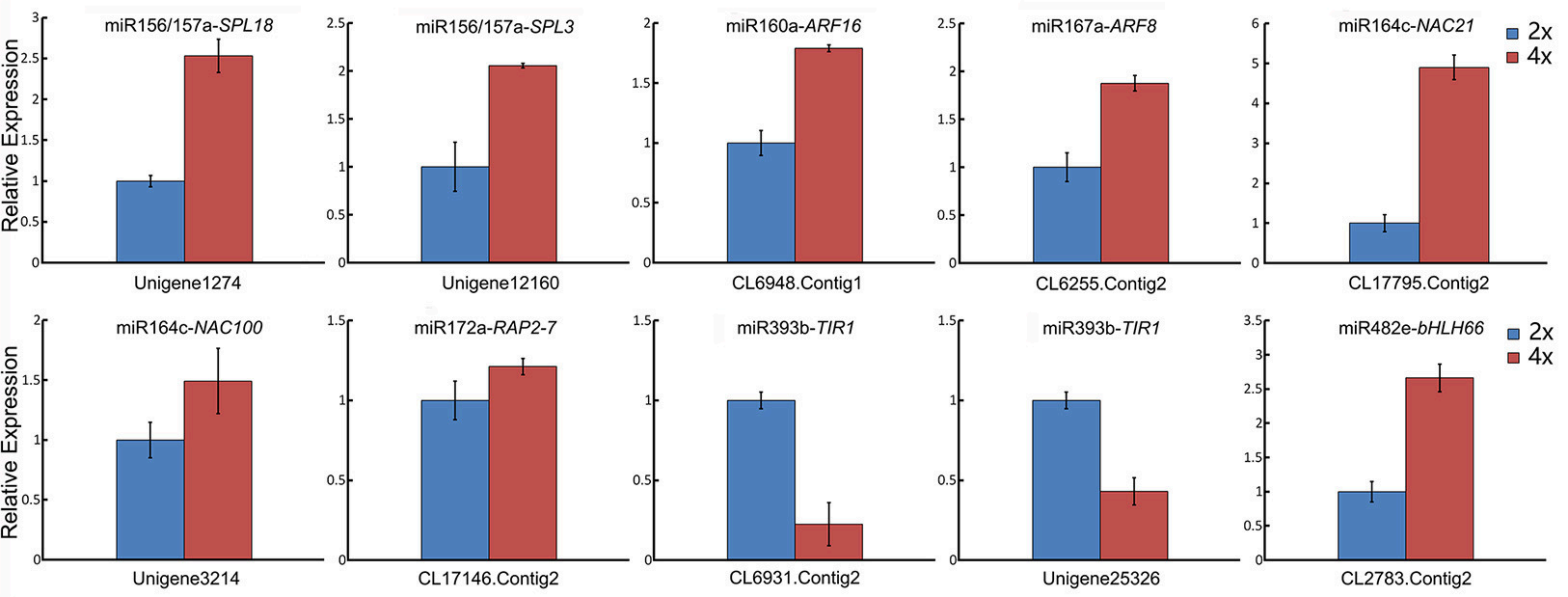
**qRT-PCR-estimated abundance of transcript of selected gene targets involved in growth and development and associated with miRNAs in the 2x and 4x form of *C. nankingense***. Each bar shows the mean ± SE of a triplicated assay.

When a series of qRT-PCRs was performed to validate the differential abundance of ten of the known miRNAs and their targets, full consistency was achieved with the sequencing outcome (Figure 6). The expression of miRNAs and their potential targets involved in growth and development were investigated, 16 known miRNAs were identified in 4x *vs*. 2x (Figure 7). In addition, 10 potential targets included *SPL* (Unigene1274, Unigene12160), *ARF* (CL6948.Contig1, CL6255.Contig2), *NAC* (CL17795.Contig2, Unigene3214), *AP2* (CL17146.Contig2), *TIR1* (CL6931.Contig2, Unigene25326), and *bHLH* (CL2783.Contig2) were identified in diploid and autotetraploid (Figure 8, Table S7).

## Discussion

Since the majority of angiosperm species are recognizably polyploid or, like both maize and *A. thaliana*, have decayed from a polyploid progenitor (“cryptic” polyploids), it has been concluded that polyploidization has been a major driver of plant evolution (Adams and Wendel, 2005). Evidence is accumulating that the activity of miRNAs is important in shaping newly polyploidized genomes (Vazquez et al., 2008; Haa et al., 2009). Thus, the intention here was to explore how they influence the growth and development of the autotetraploid form of *C. nankingense*.

### Autopolyploidization has immediate effect on the range of miRNAs produced

The founder pool of miRNAs, at least in monocotyledonous species, is very ancient, and the pace of birth and death of conserved miRNAs is slow (Abrouk et al., 2012). In both the 2x and 4x forms of *C. nankingense*, the known miRNA distribution was dominated by molecules in the length range 20–25 nt (Figure S1, Table S8), 22 nt (56.17%), and 25 nt (26.0%) known miRNAs displayed the predominant population in the 2x *C. nankingense*, but 21 nt (50.93%) and 24 nt (24.84%) known miRNAs dominated in the 4x *C. nankingense*. In the *A. thaliana* diploid a variety of miRNAs (21–25 nt in length) is biologically significant (Vazquez et al., 2008), it is mainly the 21 nt group which is responsible for differential gene expression in *Arabidopsis* spp. allopolyploids (Haa et al., 2009). However, in tomato hybrids, Li J. et al. (2014) have shown that it is the 24 nt group which most strongly influences gene expression. At least four *Dicer-like* (*DCL*)-encoded enzymes (DCL1–DCL4) catalyze the formation of miRNAs in *Arabidopsis* (Kasschau et al., 2007), DCL1 have an important role in miRNA accumulation (Kurihara and Watanabe, 2004). Those of length 21 nt and 23–25 nt are generated by DCL1 and DCL3 (Kurihara and Watanabe, 2004; Vazquez et al., 2008). The accumulation of 23–25 nt miRNAs has been correlated with the organ-specific expression of *DCL* (Vazquez et al., 2008). When the transcription of the various Dicer homolog *DCLs* was quantified in both diploid and autotetraploid *C. nankingense*, there was no evidence of any up- or down-regulation (Figure S2). Thus, autopolyploidization is thought likely to have a significant immediate effect on the distribution of miRNAs.

### The effect of autopolyploidization on miRNA abundances

Despite there being little evidence for changes induced by autopolyploidization in the range of miRNAs present, there was a distinct effect on the abundance of some of them. Thus, the copy number of 72 known and 35 novel miRNAs was higher in the 4x than in the 2x form of *C. nankingense*. Of these, 49 known and 25 novel miRNAs were not detected in the diploid, but were highly abundant in the autotetraploid (Tables S4, S6). At the same time, the abundance of 71 known and 42 novel miRNAs was reduced by autopolyploidization; of these 52 known and 28 novel ones were undetectable in the autotetraploid (Tables S4, S6). In some *Brassica* spp. allopolyploids, it has been claimed that genome redundancy may reflect the increased frequency of changes in miRNA abundance (Shen et al., 2014). In wheat, the proportion of sRNAs classifiable as miRNAs increases with ploidy level (Kenan-Eichler et al., 2011), while according to Li A. et al. (2014), in wheat hexaploids, the abundance of many miRNAs does not reflect additivity. Autopolyploidization is also relatively common in plants, its immediate result is genetic redundancy and process genome flexibility (Parisod et al., 2010). It is suggested that autopolyploidization as well as allopolyploidization has a considerable effect on the population of miRNAs, and thus on gene expression. In addition, miRNAs are associated with non-additive expression of target genes, the expression variation of miRNAs can lead to changes in gene expression, growth vigor, and adaptation (Chen, 2007; Ha et al., 2009). The accumulations of miRNAs contribute to their targets diversity resulting in different growth vigor in autotetraploid relative to diploid. This process could confer a benefit in terms of greater fitness and/or adaptation to an autopolyploid such as 4x *C. nankingense*.

### Differentially abundant miRNAs targeting genes involved in growth and development

MiRNAs are ubiquitous in both plant and animal genomes (Ha et al., 2008). In plants, miRNAs can influence a number of aspects of phenotype, growth, and development and the stress response (Dugas and Bartel, 2004; Zhang et al., 2006). Several of the potential miRNA target genes have been related to the regulation of growth and development (Table 4, Figure 8). miR156a/157a, which was less abundant in the autotetraploid than in the diploid, targets five members of *SQUAMOSA PROMOTER BINDING PROTEIN-LIKE* (*SPL*) family, genes which play fundamental roles in growth, development and metabolism (Lei et al., 2016) and may associate with the vigor of the autotetraploid. miR160a and miR167a interact with *AUXIN RESPONSE FACTOR* (*ARF*) genes, the products of which showed more abundant in autotetraploid relative to diploid, regulate a variety of physiological processes, especially plant growth and development (Guilfoyle and Hagen, 2007). MiR393b negatively mediate the gene *TRANSPORT INHIBITOR RESPONSE 1* (*TIR1*), the product of which limits the plant's sensitivity to auxin, with a knock-on effect on development (Chen et al., 2011). However, the gene of *TIR1* down-regulated after autopolyploidization, suggesting that auxin may contribute to the vigor of autotetraploid *C. nankingense*. miR172a targets three members of *APETALA2* (*AP2*) genes and one, the former is a class of transcription factors which includes a key regulator of floral development (Riechmann and Meyerowitz, 1998; Spanudakis and Jackson, 2014), and the latter involved in stress responses, secondary metabolism, growth and developmental programs (Licausi et al., 2013). The transcription factor is important for environmental stimuli to enhance the adaption of autotetraploid *C. nankingense*. The potential target of miR419 and miR1098 is the *UPL* family of genes encoding E3 ubiquitin-protein ligases, which are an important component of ubiquitination, a post-translational modification delivering plasticity and environmental adaptation (Stone and Callis, 2007). Finally, miR482e interacts with a member of the bHLH transcription factor family, many members of which act as key regulators of gene expression (Toledo-Ortiz et al., 2003). Taken together, miRNAs play important roles in plant growth and development in *C. nankingense* by negatively mediating targets. The involvement of miRNAs in such genes may explain why the autotetraploid form of *C. nankingense* is more vigorous than its diploid progenitor.

## Conclusion

Here, a comparison was drawn between the miRNA content of diploid and autotetraploid *C. nankingense* with the intention of exploring the basis of the latter's more vigorous phenotype. Thus autopolyploidization seems likely to have had an immediate effect the distribution of miRNAs, and it have a marked effect on the abundance of many of the miRNAs, including a number which target genes of known importance to growth and development in other plants. The suggestion is that miRNAs may well-contribute to the greater vigor displayed by the autotetraploid form of *C*. *nankingense*.

## Author contributions

BD and JJ contributed to bioinformatics analysis and writing of the manuscript. HW, ZG, and FC conceived of the study, participated in its design, and contributed to revisions of the manuscript. WF and TL participated in experiment materials preparation. YC and SC helped with the RNA extraction and real-time PCR, all authors read and approved the final manuscript.

## Funding

Fundamental Research Funds for the National Nature Science Foundation of China (31272203), the Central Universities (KYTZ201401), “Programs of Innovation and Entrepreneurship Talents” of Jiangsu Province Project Funded by the Priority Academic Program Development of Jiangsu Higher Education Institutions.

### Conflict of interest statement

The authors declare that the research was conducted in the absence of any commercial or financial relationships that could be construed as a potential conflict of interest.

## Supplementary material

Figure S1 **The length distribution of miRNAs in 2x and 4x *C. nankingense***.

Figure S2 **The expression of *DCLs* in the 2x and 4x form of *C. nankingense***. Each bar shows the mean ± SE of a triplicated assay. The results showed insignificant differences between the 2x and 4x form of *C. nankingense*. Significant differences were determined by *t*-test (*P* < 0.05).

Table S1 **The full set and the set of unique sRNAs present in the diploid and autotetraploid forms of *C. nankingense***.

Table S2 **Variation in the distribution of sRNAs present in the diploid and autotetraploid forms of *C. nankingense***.

Table S3 **The count and sequences of known miRNAs in the diploid and autotetraploid forms of *C. nankingense***.

Table S4 **The differential abundance of known miRNAs in the diploid and autotetraploid forms of *C. nankingense***.

Table S5 **The count and sequences of novel miRNAs in the diploid and autotetraploid forms of *C. nankingense***.

Table S6 **The differential abundance of novel miRNAs in the diploid and autotetraploid forms of *C. nankingense***.

Table S7 **Primer sequences used to amplify target genes by qRT-PCR**.

Table S8 **The distribution of miRNA in 2x and 4x form *C. nankingense***.

Sheet 1 **The predicted targets of miRNAs which differed in abundance between the diploid and autotetraploid forms of *C. nankingense***.

Sheet 2 **Gene ontology classification of the targets of miRNAs which differed in abundance between the diploid and autotetraploid forms of *C. nankingense***.

Sheet 3 **KEGG pathway annotation of the targets of miRNAs which differed in abundance between the diploid and autotetraploid forms of *C. nankingense***.
