# Supplementary figures and images for: miRNAs Are Involved in Determining the Improved Vigor of Autotetrapoid Chrysanthemum nankingense
Source: Front Plant Sci. 2016 Sep 28;7:1412. doi: 10.3389/fpls.2016.01412 (PMC5039203; doi:10.3389/fpls.2016.01412)

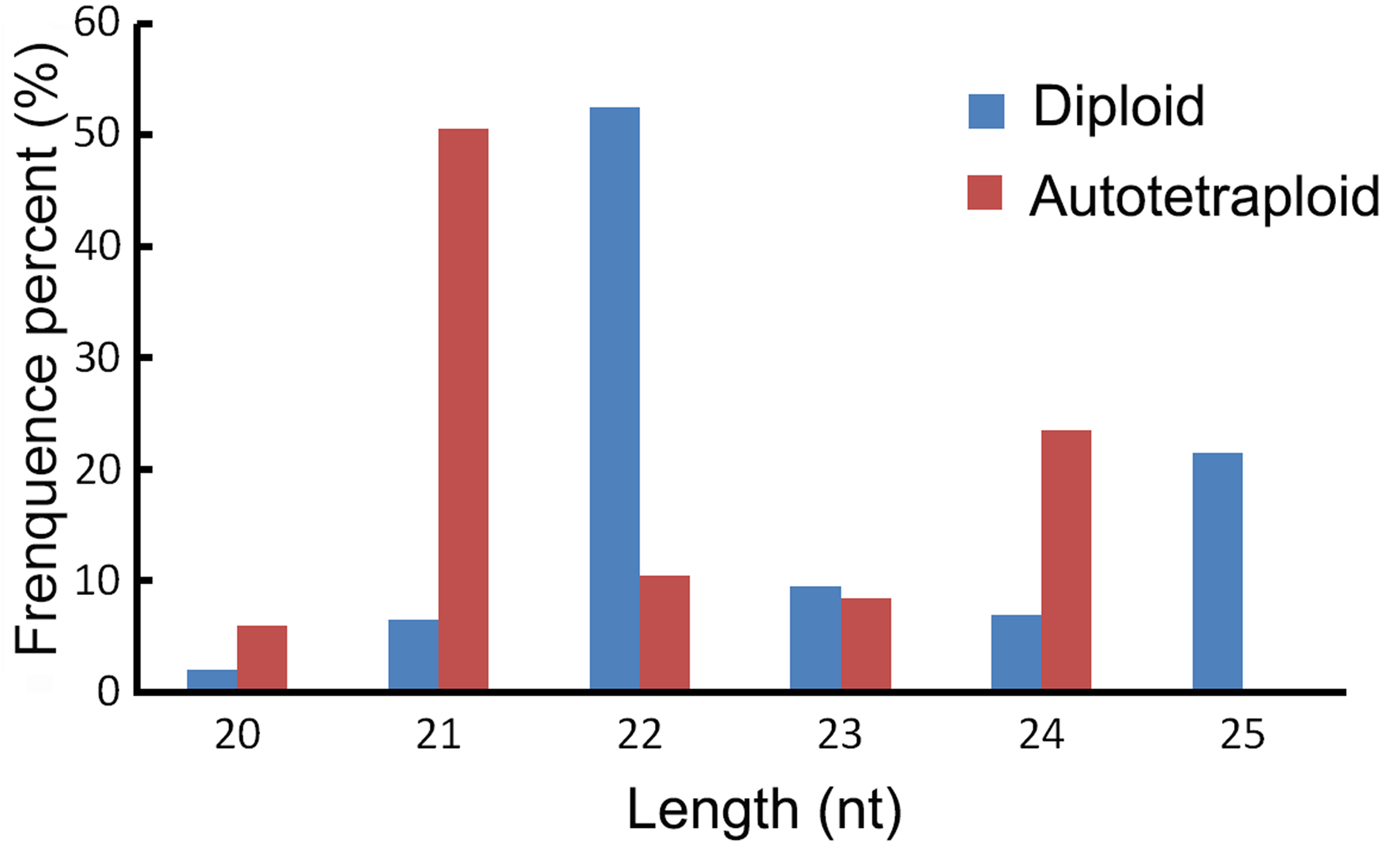

Supplement: Figure S1 — The length distribution of miRNAs in 2x and 4x C. nankingense. [file Image1.tif]

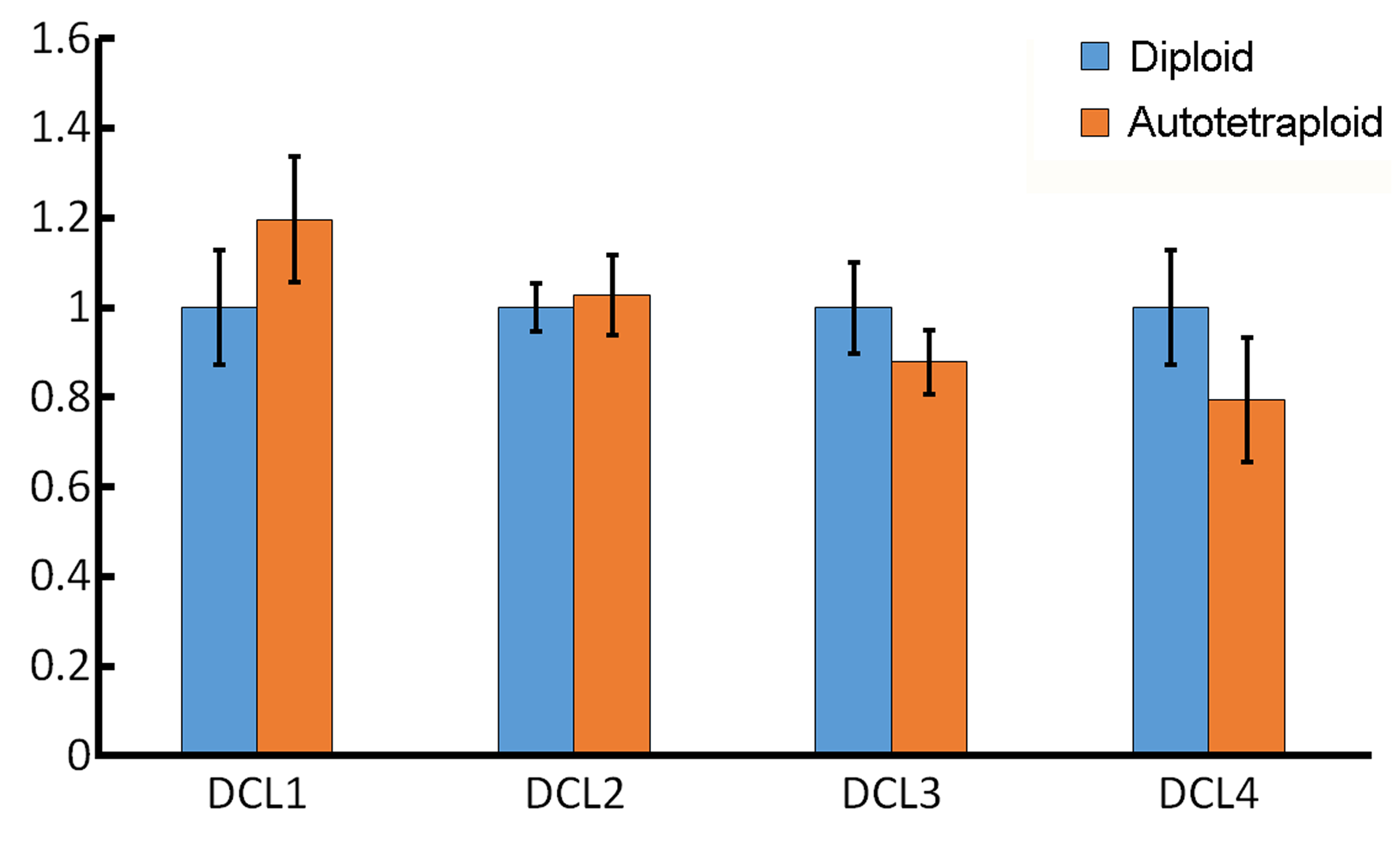

Supplement: Figure S2 — The expression of DCLs in the 2x and 4x form of C. nankingense. Each bar shows the mean ± SE of a triplicated assay. The results showed insignificant differences between the 2x and 4x form of C. nankingense. Significant differences were determined by t-test (P < 0.05). [file Image2.tif]
