# Supplementary material for: miRNAs Are Involved in Determining the Improved Vigor of Autotetrapoid Chrysanthemum nankingense
Source: Front Plant Sci. 2016 Sep 28;7:1412. doi: 10.3389/fpls.2016.01412 (PMC5039203; doi:10.3389/fpls.2016.01412)
Supplement: Table S1 — The full set and the set of unique sRNAs present in the diploid and autotetraploid forms of C. nankingense. [file Table1.docx]

**Table S1** **The full set and the set of unique sRNAs present in the diploid and autotetraploid forms of *C. nankingense***

|  | Total sRNAs | Percent (%) | Unique sRNAs | Percent (%) |
| --- | --- | --- | --- | --- |
| Total | 38,141,274 | 100.00% | 10,457,666 | 100.00% |
| 4x & 2x | 23,769,940 | 62.32% | 1,049,162 | 10.03% |
| 4x specific | 7,667,538 | 20.10% | 4,864,658 | 46.52% |
| 2x specific | 6,703,796 | 17.58% | 4,543,846 | 43.45% |
