# Supplementary material for: miRNAs Are Involved in Determining the Improved Vigor of Autotetrapoid Chrysanthemum nankingense
Source: Front Plant Sci. 2016 Sep 28;7:1412. doi: 10.3389/fpls.2016.01412 (PMC5039203; doi:10.3389/fpls.2016.01412)
Supplement: Table S2 — Variation in the distribution of sRNAs present in the diploid and autotetraploid forms of C. nankingense. [file Table2.docx]

**Table S2 Variation in the distribution of sRNAs present in the diploid and autotetraploid forms of *C. nankingense***

| Category | Diploid | | Autotetraploid | |
| --- | --- | --- | --- | --- |
|  | **Unique sRNAs** | **Total sRNAs** | **Unique sRNAs** | **Total sRNAs** |
| Total sRNAs | 5,593,008 | 17,762,959 | 5,913,820 | 20,378,315 |
| miRNA | 19,749 | 1,870,037 | 22,690 | 1,526,838 |
| rRNA | 37,201 | 309,304 | 24,121 | 312,743 |
| snRNA | 1,888 | 3,717 | 1,961 | 4,249 |
| snoRNA | 500 | 1,016 | 582 | 1,108 |
| tRNA | 9,025 | 224,567 | 4,808 | 155,098 |
| Unannotated | 5,524,645 | 15,354,318 | 5,859,658 | 18,378,279 |
