# Supplementary material for: miRNAs Are Involved in Determining the Improved Vigor of Autotetrapoid Chrysanthemum nankingense
Source: Front Plant Sci. 2016 Sep 28;7:1412. doi: 10.3389/fpls.2016.01412 (PMC5039203; doi:10.3389/fpls.2016.01412)
Supplement: Table S7 — Primer sequences used to amplify target genes by qRT-PCR. [file Table7.docx]

**Table S10. Primer sequences used to amplify genes by qRT-PCR**

| Gene ID | Anotation | Sequence |
| --- | --- | --- |
| Unigene1274 | *SPL* | ACCACTTGATTCCGTAAGCATT |
|  |  | AACTTCCTCCAACACCGACC |
| Unigene12160 | *SPL* | GCGTGGGGACTGTGAACTAAG |
|  |  | TCTGGTGAAGCGATGATTGG |
| CL6948.Contig1 | *ARF* | CATCCACATCAACCGAAGAAAT |
|  |  | CGGGCAAGTACGCCAGAGT |
| CL6255.Contig2 | *ARF* | GCATCGAGTGTTGGAAGCAG |
|  |  | CTAATCGGCAATCGTGGGT |
| CL17795.Contig2 | *NAC* | AGGGCACTTGCTGCTGATTAT |
|  |  | GGGTGCTATGTAGAGTGTTTTGC |
| Unigene3214 | *NAC* | GGATGTTGATTGGATTGGAGAA |
|  |  | CCGCAAAGAATGAATGGGT |
| CL17146.Contig2 | *AP2* | GCACCTCTTGACTCACTGGAAA |
|  |  | ACTGTCTGCTGATGGTGATGGA |
| CL6931.Contig2 | *TIR1* | CACCCCAATCATCAGGCACA |
|  |  | CCACAAAGACCGCAGCTCAA |
| Unigene25326 | *TIR1* | CAGCCCCTTCATCTATAACTTCA |
|  |  | GAGACTATGCGATCCCTTTGG |
| CL2783.Contig2 | *bHLH* | CTCATCTAGCATTGAAGCCTTGT |
|  |  | AGTTTCCATTTCCTCCGACC |
| CL5553.Contig1_All | *DCL1* | ATTGGGAGTGTTTCTGGGGTC |
|  |  | CTCATTTGGACTGGGAGACTGT |
| Unigene19517_All | *DCL2* | CATCCCCAAGAAACTCTAAACG |
|  |  | GAAACTTCACATTGCGACCAG |
| Unigene4495_All | *DCL3* | TGCTGGCAGTAGATAGAACGATT |
|  |  | AGGACAACATCTATTGCTGCTCA |
| CL2877.Contig1_All | *DCL4* | CGTGCCCTAAAACTGGAAAGAA |
|  |  | CGAGCAAAACGATGATGATGT |
