# Supplementary material for: miRNAs Are Involved in Determining the Improved Vigor of Autotetrapoid Chrysanthemum nankingense
Source: Front Plant Sci. 2016 Sep 28;7:1412. doi: 10.3389/fpls.2016.01412 (PMC5039203; doi:10.3389/fpls.2016.01412)
Supplement: Table S8 — The distribution of miRNA in 2x and 4x form C. nankingense. [file Table8.docx]

**Table S11 The distribution of miRNA in 2x and 4x form *C. nankingense***

| Libraries | Distribution | | | | | |
| --- | --- | --- | --- | --- | --- | --- |
|  | 20 nt | 21 nt | 22 nt | 23 nt | 24 nt | 25 nt |
| Diploid | 3 (1.85%) | 8 (6.04%) | 91 (56.17%) | 16 (9.88%) | 9 (5.56%) | 35 (21.60%) |
| Autotetraploid | 7 (4.35%) | 82 (50.93%) | 17 (10.56%) | 15 (9.32%) | 40 (24.84%) | 0 (0%) |
